# Platelet-Rich Plasma Injection Associated With Microtenotomy in Lateral Epicondylitis – is a Tendon Tear Associated with the Therapeutic Response?

**DOI:** 10.7759/cureus.22425

**Published:** 2022-02-21

**Authors:** Joana Martins, Igor S Neto, Ana F Gonçalves, Adriana Pereira, Mariana Santiago, Inês Ferro, Tiago Lopes, José Luís Carvalho

**Affiliations:** 1 Physical Medicine and Rehabilitation, Centro Hospitalar e Universitário de Coimbra, Coimbra, PRT; 2 Physical Medicine and Rehabilitation, Centro de Reabilitação do Norte, Vila Nova de Gaia, PRT; 3 Physical Medicine and Rehabilitation, Centro de Medicina de Reabilitação de Alcoitão, Alcoitão, PRT; 4 Physical Medicine and Rehabilitation, Centro Hospitalar Universitário de São João, Porto, PRT; 5 Physical Medicine and Rehabilitation, Centro de Medicina de Reabilitação da Região Centro - Rovisco Pais, Tocha, PRT; 6 Intervention and Musculoskeletal Rehabilitation Unit, Centro de Reabilitação do Norte, Vila Nova de Gaia, PRT

**Keywords:** ultrasound-guided, pain, tenotomy, platelet-rich plasma, epicondylitis

## Abstract

Objective

Ultrasound-guided platelet-rich plasma (PRP) injections, as well as needle tenotomy, are becoming increasingly popular in the treatment of epicondylitis. Whether ultrasound (US) findings predict the clinical benefit of these techniques is unclear at the moment. This study aimed to investigate the relationship between the presence of tendon tear assessed by US and the therapeutic response of the PRP injection following needle microtenotomy in patients with epicondylitis.

Methodology

This is a retrospective observational study. Twenty-six patients with chronic (>three months) lateral epicondylitis recalcitrant to conservative treatment or corticosteroid injection. Patients underwent US-guided microtenotomy followed by PRP injection. Data regarding gender, age, US findings at baseline, and numeric pain rating scale (NPRS) scores before and after intervention were collected. Pain improvement rates were calculated at several follow-up time points, namely one, three, six, and 12 months post-intervention. Results are stated as mean ± standard deviation.

Results

At the time of intervention, the mean age was 47.6±6.5 years, and 57.7% of patients were men. Overall, the mean initial NPRS score was 7.5±1.2, and there were no statistically significant differences in mean initial NPRS scores between the groups with or without tendon tear on the US imaging. The mean improvement rate at one, three, and six months was similar between patients with and without tendon tear. However, a statistically significant difference was observed at 12 months (73.1±37.6% vs. 16.0±21.9, p=0.029).

Conclusions

Patients with tendon tear demonstrated a higher pain improvement rate at 12 months follow-up. This finding could predict the clinical response to this technique, thus allowing a better selection of the candidates.

## Introduction

Lateral humeral epicondylitis is a common source of lateral elbow pain, which was first described by Runge in 1873, and is generally accepted as a condition related to repetitive microtrauma [1,2]. Despite its exact pathophysiologic mechanisms are still unclear, an accumulating body of evidence suggests an underlying process of degenerative tendinosis characterized by the absence of inflammatory cells and the presence of fibroblasts, vascular hyperplasia, and unstructured collagen instead of a continuous inflammatory process [3-5].

Several therapeutic strategies have been described, namely analgesic drugs, physiotherapy, elbow supports, shockwave therapy, corticosteroid injections, acupuncture, and surgery. However, there is still no generally accepted treatment, and the more is known about its pathophysiology, the more treatments that stimulate tissue regeneration become popular [6,7].

Percutaneous needle tenotomy, also known as tendon fenestration or barbotage, consists of the fenestration of the tendinopathic tissue with a needle to induce bleeding and the release of growth factors, thus converting a chronic into an acute injury with high healing potential. Similarly, autologous platelet-rich plasma (PRP) injections aim to stimulate the healing process by promoting the release of active cytokines by the platelets when injected into the area of tendinopathy [7,8].

Despite several authors have been reporting good results with these techniques, either isolated or used in association, in the treatment of tendinopathic conditions [7-15], little is known about the predictors of its therapeutic benefit in epicondylitis. This study aimed to investigate the relationship between the presence of tendon tear assessed by ultrasound (US) and the therapeutic response of the US-guided PRP injection following needle tenotomy in patients with epicondylopathy.

## Materials and methods

This retrospective observational study included 26 consecutive patients with chronic (> three months) lateral epicondylitis with no or unsatisfactory improvement after conservative treatment or corticosteroid injection.

Clinical diagnosis and the pre-intervention US

Two experienced physicians in musculoskeletal pathology from our center performed the diagnosis, pre-intervention US, and the US-guided microtenotomy followed by PRP injection procedure to all patients. Clinical diagnosis was based on pain at the common extensor origin at the lateral humeral epicondyle, exacerbated by local palpation, forearm supination resistance, active wrist extension, and positive Cozen test. Pre-intervention US was performed with a 6-13MHz high-frequency linear probe (GE Logiq F8 L6-12-RS, General Electric Company GE, United Kingdom).

All the patients gave their consent, and the procedures in this study were in agreement with ethical standards and accordance with the 1964 Helsinki Declaration and its later amendments.

PRP preparation

For PRP preparation, 20 mL of peripheral blood were collected from all patients, and leukocyte-free PRP was prepared by single spinning at 1500 rpm for eight minutes. The plasma layer was collected under laminar flow, obtaining approximately 4mL of pure PRP, with no external activation.

US-guided microtenotomy and PRP injection

All procedures were performed in the supine position, with the elbow flexed at 120° and the forearm in pronation, following a sterile protocol. Lidocaine (2mL) was initially infiltrated in the subcutaneous tissues superficial to the lateral epicondyle via a 21-gauge needle. After that, 15-20 fenestrations were applied on the tendon by redirecting the needle in different directions, especially over the hypoechogenic tissue, followed by PRP injection in the same areas. All patients were then instructed to rest for 48 hours and avoid weight lifting and manipulation.

Data collection and outcomes

Data regarding gender, age, and US findings at baseline were collected from patients' records. Concerning US findings, patients were grouped according to the existence or absence of intratendinous tear.

The primary endpoint was the mean numeric pain rating scale (NPRS) score throughout the follow-up (one, three, six, and 12 months post-intervention). Secondary endpoints included the mean change in NPRS score from baseline, the improvement rate (%) calculated as a percentage of reduction of NPRS score from baseline and pain intensity classified as no pain (NPRS score = 0), mild (1-2), moderate (3-7) or severe (8-10) pain.

Statistical analysis

Data were presented as mean ± standard deviation (SD) for continuous variables or as frequencies and percentages for categorical variables, as n indicates the number of patients. Normality of distribution for continuous variables was assessed with the Shapiro-Wilk test. Univariate analysis was performed with (i) the Student's t-test or the Mann-Whitney U test for continuous variables, according to the normality assessment, or (ii) the 𝜒2 test or the Fisher's exact test for categorical variables, as appropriate. A p-value <0.05 was considered as the threshold for statistically significant differences. Statistical analysis was carried out with IBM SPSS Statistics version 19.0.0 (IBM Inc., Armonk, NY, USA), and graphs were prepared with ggplot2 package for R using RStudio (version 1.4.1106).

## Results

Baseline characteristics of the population

A total of 26 patients were included in this study. Table 1 displays baseline patient characteristics according to gender. The average age was 47.6±6.5 years. The majority of the patients were male (57.7%), and right elbow epicondylitis was more prevalent (57.7%) than left. Tendon tear was present in 17 patients (65.4%).

**Table 1:** Baseline patient characteristics according to gender Values are presented as a number of patients (%) or as mean ± SD.

Characteristic	Total (n=26)	Female (n=11)	Male (n=15)	P-value
Age (years) ± SD	47.6±6.5	47.0±7.5	48.1±6.0	0.689
Elbow				
Left	11 (42.3)	6 (54.5)	5 (33.3)	0.426
Right	15 (57.7)	5 (45.5)	10 (66.7)	
Tear	17 (65.4)	8 (72.7)	9 (60.0)	0.683

NPRS score throughout follow-up

**Figure 1:**
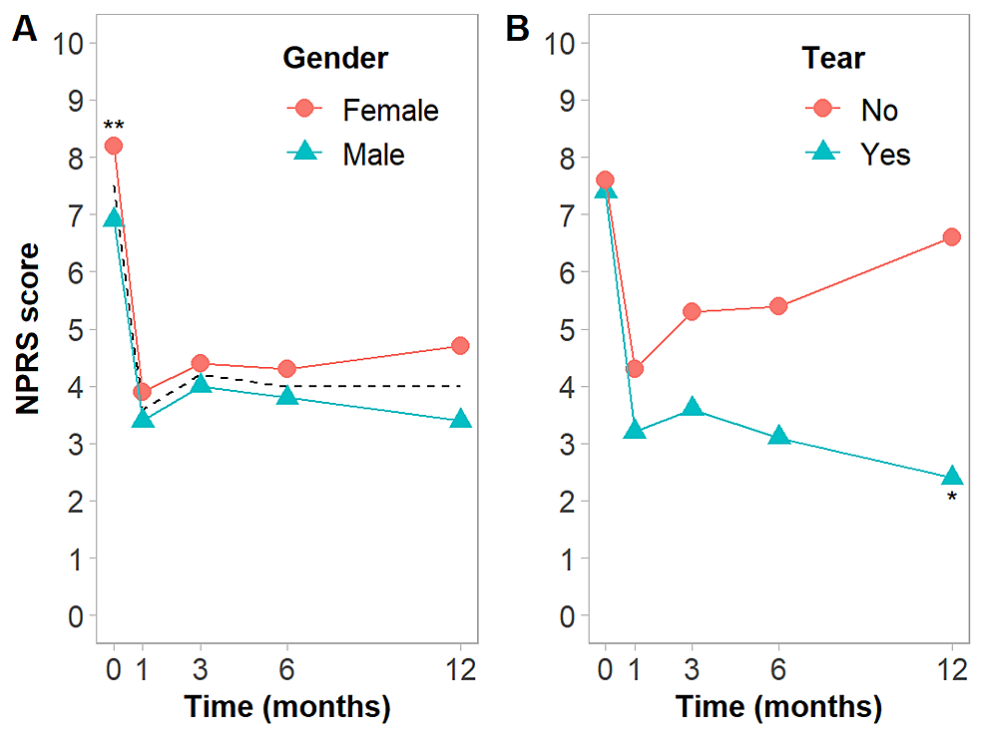
Mean NPRS score throughout follow-up according to gender (A) and the presence of tendon tear (B) on US In A, the dashed line represents overall mean values. *p<0.05, **p<0.01 NPRS - numeric pain rating scale; US - ultrasound

Overall, the mean initial NPRS score was 7.5±1.2 (Table 2), with a statistically significant difference (p=0.007) between men (6.9±0.8) and women (8.2±1.4), as seen in Figure 1A. Table 2 shows that 50% of the patients had moderate pain and 50% severe pain at baseline.

**Table 2:** NPRS score-based outcomes throughout follow-up Values are presented as a number of patients (%) or as mean ± SD. ∆ - represents change; NPRS - numeric pain rating scale.

Outcomes	Baseline (n=26)	Follow-up			
		One month (n=19)	Three months (n=19)	Six months (n=18)	12 months (n=13)
NPRS score	7.5±1.2	3.6±2.7	4.2±3.2	4.0±3.0	4.0±3.5
∆ from baseline	-	-3.9±2.9	-3.2±3.0	-3.3±2.6	-3.7±3.0
Improvement rate (%)	-	50.3±36.0	43.7±40.1	46.9±38.6	51.2±42.7
Pain intensity					
No pain	-	1 (5.3)	3 (15.8)	2 (11.1)	4 (30.8)
Mild	-	8 (42.1)	4 (21.1)	6 (33.3)	2 (15.4)
Moderate	13 (50.0)	7 (36.8)	8 (42.1)	7 (38.9)	4 (30.8)
Severe	13 (50.0)	3 (15.8)	4 (21.1)	3 (16.7)	3 (23.1)

Mean NPRS score, mean change in NPRS score from baseline, mean improvement rate, and pain intensity at one, three, six, and 12 months are presented in Table 2. Six patients, three females and three males (23.1%), were refractory to treatment, as no changes in NPRS score were observed between baseline and throughout the follow-up.

No significant differences were observed between men and women at the evaluated time points of follow-up, although scores tended to be higher in women (Table 3).

**Table 3:** NPRS score-based outcomes according to gender Values are presented as a number of patients (%) or as mean ± SD. NPRS - numeric pain rating scale

Outcomes	Female	Male	P-value
NPRS score			
Baseline	8.2±1.4	6.9±0.8	0.007
One month	3.9±3.1	3.4±2.5	0.697
Three months	4.4±3.4	4.0±3.1	0.770
Six months	4.3±3.1	3.8±3.1	0.752
12 months	4.7±3.1	3.4±4.0	0.611
Improvement rate (%)			
One month	50.6±38.8	50.0±35.8	0.971
Three months	45.0±40.0	42.5±42.3	0.934
Six months	51.7±33.0	44.6±42.3	0.738
12 months	46.7±33.4	55.0±51.7	0.769

Although patients with tear showed a trend towards greater improvement than patients without tear (Figure 1B), this difference was only statistically significant at 12 months in this population (Table 4). No differences were identified in baseline clinical characteristics between patients with tear and patients without tear.

**Table 4:** NPRS score-based outcomes according to the presence of tear assessed by US Values are presented as a number of patients (%) or as mean ± SD. NPRS - numeric pain rating scale; US - ultrasound; ∆ - represents change

Outcomes	Tear	No tear	P-value
NPRS score			
Baseline	7.4±1.3	7.6±1.2	0.785
One month	3.2±2.4	4.3±3.4	0.790
Three months	3.6±3.2	5.3±3.0	0.251
Six months	3.1±2.9	5.4±2.8	0.113
12 months	2.4±3.4	6.6±1.7	0.044
∆ from baseline			
One month	-4.1±2.5	-3.5±3.9	0.823
Three months	-3.9±3.0	-1.9±2.5	0.182
Six months	-4.2±2.4	-2.0±2.6	0.090
12 months	-5.1±2.6	-1.4±1.9	0.025
Improvement rate (%)			
One month	53.8±31.5	42.5±46.7	0.790
Three months	52.5±39.6	28.6±39.1	0.181
Six months	59.1±35.8	27.9±37.2	0.087
12 months	73.1±37.6	16.0±21.9	0.029

As this is a retrospective study, not all patients had a 12-month follow-up time. Thus, in order to validate these results, a subset analysis of patients with 12 months follow-up was performed, which confirmed the results shown above (Table 5).

**Table 5:** NPRS score-based outcomes according to the presence of tear assessed by US in the validation subset (patients with 12-month follow-up) Values are presented as the number of patients (%) or as mean ± SD. NPRS - numeric pain rating scale; US - ultrasound; ∆ - represents change

Outcomes	Tear	No tear	P-value
NPRS score			
Baseline	7.5±1.2	8.0±1.2	0.482
One month	2.8±3.2	1.3±0.6	0.423
Three months	2.4±3.4	4.7±3.2	0.247
Six months	2.9±3.0	5.5±3.1	0.192
12 months	2.4±3.4	6.6±1.7	0.044
∆ from baseline			
One month	-4.3±2.6	-7.0±1.0	0.137
Three months	-5.1±3.0	-2.7±2.9	0.250
Six months	-4.6±2.3	-2.5±3.0	0.196
12 months	-5.1±2.6	-1.4±1.9	0.025
Improvement rate (%)			
One month	63.3±35.0	85.0±5.0	0.337
Three months	71.4±38.5	38.3±40.4	0.203
Six months	65.6±33.7	31.3±40.5	0.149
12 months	73.1±37.6	16.0±21.9	0.029

## Discussion

The pathophysiology of epicondylitis is related to chronic inflammation, characterized by the presence of cellular degenerative processes and microvascular dysregulation. Thus, disorganization of the tendon healing process is predominantly observed, and the presence of inflammatory cells is only characteristic of the initial phases of this clinical condition. Hence, the available evidence about the pathophysiological mechanisms of epicondylitis increasingly supports the use of strategies that promote tissue regeneration instead of therapies with a predominant anti-inflammatory action [16].

In this way, the injection of PRP has been used with the aim of promoting healing in epicondylitis and other clinical entities with tendon involvement, with significant pain reduction both in the short and long term [17]. US-guided percutaneous needle tenotomy has also showed sustained pain improvement over time, considered as a minimally invasive alternative to the surgical release of the common tendon of the extensors [18-19]. Currently, there is no data supporting the use of one technique over the other, and the association of PRP injection and percutaneous microtenotomy is also little described in the literature. Although this association has been shown to be effective in pain reduction, its real impact in comparison to the isolated techniques has not yet been studied [13-15, 20]. Thus, the real efficacy of these techniques remains unclear due to the lack of quality evidence [6].

In our sample, right epicondylitis was more prevalent than left (n=15, 57.7% vs. n=11, 42.3%), which is not surprising as epicondylitis is associated with overuse and is more frequently observed on the dominant side. The mean age of the patients studied was 47.6±6.5 years, which was also consistent with the literature [21]. On the contrary, the majority of patients were male (n=15, 57.7%), although the female gender has been reported as a demographic factor associated with a higher probability of developing epicondylitis [22].

We found a significantly higher baseline NPRS score for women (8.2±1.4 vs. 6.9±0.8, p=0.007). Several authors have reported that men and women differ in their responses to pain, with increased pain sensitivity and more painful diseases commonly reported among women, which is consistent with these findings [23]. However, we found no relationship between gender and the therapeutic effect of PRP injection following US-guided percutaneous microtenotomy, although the male gender had been previously associated with better functional recovery post-tenotomy in one study [15], and the female gender was considered to have more benefit from injecting PRP in tendinopathy [24].

The presence of tendon tear was observed in 65.4% (n=17) of the population. No relationship has been found between the initial NPRS score and the presence of this ultrasound finding. However, the presence of tear was significantly associated with higher pain relief at 12 months, both regarding the NPRS score, difference from baseline, and improvement rate. As far as we know, only one study has explored the predictive value of US findings in response to needle tenotomy with or without PRP, with no differences being found between the groups with and without tear, regarding the outcomes evaluated [15].

In all time points evaluated, there was a statistically significant reduction in NPRS score greater than three units compared to baseline. As previously proposed, these differences suggest a clinically significant reduction in the level of pain throughout the follow-up period, so this technique can be considered effective for a period of at least 12 months [7,25].

This study has several limitations, namely the fact that it is retrospective, leading to a limitation in the information that was able to be collected; the small sample size, which may hinder the interpretation of findings and comparability with previous studies. The fact that not all patients had the same follow-up period may also be a limitation in our work. Nevertheless, this aspect was addressed by performing a subset analysis of the patients with data available for the 12-month follow-up.

## Conclusions

US-guided PRP injection following percutaneous microtenotomy was effective in the majority of the patients with epicondylitis and may be a reasonable option for the treatment of this clinical entity. Patients with tendon tear demonstrated a higher pain improvement rate at 12 months follow-up. This finding could be predictive of the clinical response to this technique, thus allowing a better selection of the candidates. Prospective and larger studies should be considered with the aim of clarifying whether the presence of tears is associated with a better therapeutic response to this technique.

## References

[1] Vaquero-Picado A, Barco R, Antuña SA (2016). Lateral epicondylitis of the elbow. EFORT Open Rev.

[2] Ma KL, Wang HQ (2020). Management of lateral epicondylitis: a narrative literature review. Pain Res Manag.

[3] Johns N, Shridhar V (2020). Lateral epicondylitis: current concepts. Aust J Gen Pract.

[4] Doran A, Gresham GA, Rushton N, Watson C (1990). Tennis elbow. A clinicopathologic study of 22 cases followed for 2 years. Acta Orthop Scand.

[5] Bishai SK, Plancher KD (2006). The basic science of lateral epicondylosis: update for the future. Tech Orthop.

[6] Shergill R, Choudur HN (2019). Ultrasound-guided interventions in lateral epicondylitis. J Clin Rheumatol.

[7] Finnoff JT, Fowler SP, Lai JK, Santrach PJ, Willis EA, Sayeed YA, Smith J (2011). Treatment of chronic tendinopathy with ultrasound-guided needle tenotomy and platelet-rich plasma injection. PM R.

[8] Martin JI, Atilano L, Bully P, Iglesias G, Merino J, Grandes G, Andia I (2019). Needle tenotomy with PRP versus lidocaine in epicondylopathy: clinical and ultrasonographic outcomes over twenty months. Skeletal Radiol.

[9] Tiwari M, Bhargava R (2013). Platelet rich plasma therapy: a comparative effective therapy with promising results in plantar fasciitis. J Clin Orthop Trauma.

[10] Peck E, Ely E (2013). Successful treatment of de Quervain tenosynovitis with ultrasound-guided percutaneous needle tenotomy and platelet-rich plasma injection: a case presentation. PM R.

[11] Scholten PM, Massimi S, Dahmen N, Diamond J, Wyss J (2015). Successful treatment of athletic pubalgia in a lacrosse player with ultrasound-guided needle tenotomy and platelet-rich plasma injection: a case report. PM R.

[12] Jacobson JA, Yablon CM, Henning PT (2016). Greater trochanteric pain syndrome: percutaneous tendon fenestration versus platelet-rich plasma injection for treatment of gluteal tendinosis. J Ultrasound Med.

[13] Boden AL, Scott MT, Dalwadi PP, Mautner K, Mason RA, Gottschalk MB (2019). Platelet-rich plasma versus Tenex in the treatment of medial and lateral epicondylitis. J Shoulder Elbow Surg.

[14] Carlier Y, Bonichon F, Peuchant A (2021). Recalcitrant lateral epicondylitis: early results with a new technique combining ultrasonographic percutaneous tenotomy with platelet-rich plasma injection. Orthop Traumatol Surg Res.

[15] Martin JI, Atilano L, Merino J (2019). Predictors of outcome following tenotomy in patients with recalcitrant epicondylopathy. PM R.

[16] Pitzer ME, Seidenberg PH, Bader DA (2014). Elbow tendinopathy. Med Clin North Am.

[17] Chen X, Jones IA, Park C, Vangsness CT (2018). The efficacy of platelet-rich plasma on tendon and ligament healing: a systematic review and meta-analysis with bias assessment. Am J Sports Med.

[18] Mattie R, Wong J, McCormick Z, Yu S, Saltychev M, Laimi K (2017). Percutaneous needle tenotomy for the treatment of lateral epicondylitis: a systematic review of the literature. PM R.

[19] Seng C, Mohan PC, Koh SB, Howe TS, Lim YG, Lee BP, Morrey BF (2016). Ultrasonic percutaneous tenotomy for recalcitrant lateral elbow tendinopathy: sustainability and sonographic progression at 3 years. Am J Sports Med.

[20] Gaspar MP, Motto MA, Lewis S, Jacoby SM, Culp RW, Lee Osterman A, Kane PM (2017). Platelet-rich plasma injection with percutaneous needling for recalcitrant lateral epicondylitis: comparison of tenotomy and fenestration techniques. Orthop J Sports Med.

[21] Walz DM, Newman JS, Konin GP, Ross G (2010). Epicondylitis: pathogenesis, imaging, and treatment. Radiographics.

[22] Sayampanathan AA, Basha M, Mitra AK (2020). Risk factors of lateral epicondylitis: a meta-analysis. Surgeon.

[23] Pieretti S, Di Giannuario A, Di Giovannandrea R, Marzoli F, Piccaro G, Minosi P, Aloisi AM (2016). Gender differences in pain and its relief. Ann Ist Super Sanita.

[24] Unlu MC, Kivrak A, Kayaalp ME, Birsel O, Akgun I (2017). Peritendinous injection of platelet-rich plasma to treat tendinopathy: a retrospective review. Acta Orthop Traumatol Turc.

[25] Hróbjartsson A, Gøtzsche PC (2010). Placebo interventions for all clinical conditions. Cochrane Database Syst Rev.

